# *catena*-Poly[[diiodidocadmium(II)]-μ-4,4′-di-4-pyridyl-2,2′-disulfanediyldipyrimidine]

**DOI:** 10.1107/S1600536809051162

**Published:** 2009-12-04

**Authors:** Hai-Bin Zhu

**Affiliations:** a School of Chemistry and Chemical Engineering, Southeast University, Nanjing 211189, People’s Republic of China, and School of Material Science and Engineering, Southeast University, Nanjing 211189, People’s Republic of China

## Abstract

In the title compound, [CdI_2_(C_18_H_12_N_6_S_2_)]_*n*_, the 4,4′-di-4-pyridyl-2,2′-disulfanediyldipyrimidine (*L*) ligand bridges two Cd^II^ centers, forming polymeric zigzag chains extending along the *b* axis. The Cd^II^ ions are coordinated by two N atoms from two *L* ligands and two iodide anions in a distorted tetra­hedral geometry.

## Related literature

For coordination polymers with 4,4′-dipyridine­disulfide, see: Horikoshi & Mochida (2006 ▶). For coordination complexes with the title ligand *L*, see: Zhu *et al.* (2009 ▶).
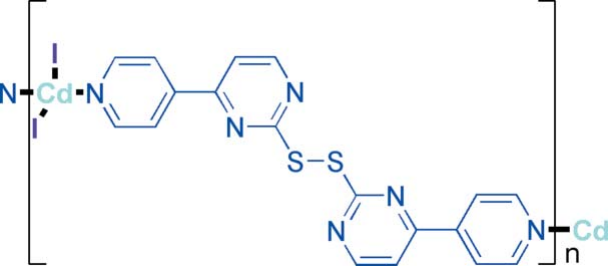


## Experimental

### 

#### Crystal data

[CdI_2_(C_18_H_12_N_6_S_2_)]
*M* *_r_* = 742.69
Triclinic, 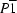

*a* = 10.0145 (7) Å
*b* = 10.7294 (8) Å
*c* = 11.7217 (9) Å
α = 93.133 (1)°
β = 109.886 (1)°
γ = 97.726 (1)°
*V* = 1166.81 (15) Å^3^
*Z* = 2
Mo *K*α radiation
μ = 3.78 mm^−1^
*T* = 298 K
0.20 × 0.15 × 0.12 mm

#### Data collection

Bruker APEXII CCD area-detector diffractometer
Absorption correction: multi-scan (*SADABS*; Bruker, 2001 ▶) *T*_min_ = 1.9, *T*_max_ = 28.9
5935 measured reflections
4037 independent reflections
3073 reflections with *I* > 2σ(*I*)
*R*_int_ = 0.015

#### Refinement

*R*[*F* ^2^ > 2σ(*F* ^2^)] = 0.027
*wR*(*F* ^2^) = 0.057
*S* = 0.99
4037 reflections
262 parameters
H-atom parameters constrained
Δρ_max_ = 0.35 e Å^−3^
Δρ_min_ = −0.35 e Å^−3^

### 

Data collection: *APEX2* (Bruker, 2007 ▶); cell refinement: *SAINT-Plus* (Bruker, 2007 ▶); data reduction: *SAINT-Plus*; program(s) used to solve structure: *SHELXS97* (Sheldrick, 2008 ▶); program(s) used to refine structure: *SHELXL97* (Sheldrick, 2008 ▶); molecular graphics: *SHELXTL* (Sheldrick, 2008 ▶); software used to prepare material for publication: *SHELXTL*.

## Supplementary Material

Crystal structure: contains datablocks I, global. DOI: 10.1107/S1600536809051162/cv2663sup1.cif

Structure factors: contains datablocks I. DOI: 10.1107/S1600536809051162/cv2663Isup2.hkl

Additional supplementary materials:  crystallographic information; 3D view; checkCIF report

## Figures and Tables

**Fig. 1.**
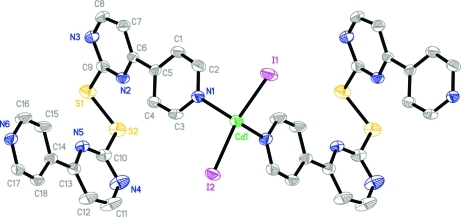
The coordination environment of the CdII center in the title compound with 30% probability displacement ellipsoids. Unlabelled atoms are related with the labelled ones by symmetry operation (x, y + 1, z). H atoms omitted for clarity.

## supplementary crystallographic information

### Comment

Over past few years, organic aromatic disulfide has received considerable attention due to both its conformational flexibility and axial chirality (Horikoshi *et al.*, 2006). In our previous study, we have reported two one-dimensional Zn^II^ and Fe^II^ coordination polymers with the ligand  *L* ( = 2, 2'-dithiobis(4-pyridin-4-ylpyrimidine) (Zhu *et al.*, 2009). Herein, we report new one-dimensional Cd^II^ coordination chain generated by althernative linking of Cd center and ligand *L*.

The Cd^II^ ion in the title complex adopts a tetrahedral coordination geometry completed by two N atoms from two ligands *L* [Cd1—N1 2.287 (3) Å; Cd1—N6 2.289 (4) Å] and two I anions [I1—Cd1 2.6938 (4) Å; I2—Cd1 2.6962 (4) Å]. In *L*,  the C—S—S—C torsion angle is 84.1 (2)°.

### Experimental

Slowly added is the CH_2_Cl_2_ (5 ml) solution of ligand *L* (0.1 mmol) into the CdI_2_ (0.1 mmol) solution in methanol (10 ml). The mixture was kept on standing for three days to give single crystals suitable for X-ray diffraction analysis.

### Refinement

All H atoms were positioned geometrically and allowed to ride on their parent atoms, with C—H = 0.93 Å and *U*iso(H) = 1.2*U*eq(C).

### Crystal data

**Table:**

[CdI_2_(C_18_H_12_N_6_S_2_)]	*Z* = 2
*M**_r_* = 742.69	*F*(000) = 696
Triclinic, *P*1	*D*_x_ = 2.114 Mg m^−^^3^
Hall symbol: -P 1	Mo *K*α radiation, λ = 0.71073 Å
*a* = 10.0145 (7) Å	Cell parameters from 4037 reflections
*b* = 10.7294 (8) Å	θ = 2.3–25.5°
*c* = 11.7217 (9) Å	µ = 3.78 mm^−^^1^
α = 93.133 (1)°	*T* = 298 K
β = 109.886 (1)°	Block, colourless
γ = 97.726 (1)°	0.20 × 0.15 × 0.12 mm
*V* = 1166.81 (15) Å^3^	

### Data collection

**Table:**

Bruker APEXII CCD area-detector diffractometer	4037 independent reflections
Radiation source: fine-focus sealed tube	3073 reflections with *I* > 2σ(*I*)
graphite	*R*_int_ = 0.015
φ and ω scans	θ_max_ = 25.0°, θ_min_ = 1.9°
Absorption correction: multi-scan (*SADABS*; Bruker, 2001)	*h* = −7→11
*T*_min_ = 1.9, *T*_max_ = 28.9	*k* = −12→12
5935 measured reflections	*l* = −13→13

### Refinement

**Table:**

Refinement on *F*^2^	Primary atom site location: structure-invariant direct methods
Least-squares matrix: full	Secondary atom site location: difference Fourier map
*R*[*F*^2^ > 2σ(*F*^2^)] = 0.027	Hydrogen site location: inferred from neighbouring sites
*wR*(*F*^2^) = 0.057	H-atom parameters constrained
*S* = 0.99	*w* = 1/[σ^2^(*F*_o_^2^) + (0.0238*P*)^2^] where *P* = (*F*_o_^2^ + 2*F*_c_^2^)/3
4037 reflections	(Δ/σ)_max_ = 0.001
262 parameters	Δρ_max_ = 0.35 e Å^−^^3^
0 restraints	Δρ_min_ = −0.35 e Å^−^^3^

### Special details

**Table:**

Geometry. All e.s.d.'s (except the e.s.d. in the dihedral angle between two l.s. planes) are estimated using the full covariance matrix. The cell e.s.d.'s are taken into account individually in the estimation of e.s.d.'s in distances, angles and torsion angles; correlations between e.s.d.'s in cell parameters are only used when they are defined by crystal symmetry. An approximate (isotropic) treatment of cell e.s.d.'s is used for estimating e.s.d.'s involving l.s. planes.
Refinement. Refinement of *F*^2^ against ALL reflections. The weighted *R*-factor *wR* and goodness of fit *S* are based on *F*^2^, conventional *R*-factors *R* are based on *F*, with *F* set to zero for negative *F*^2^. The threshold expression of *F*^2^ > σ(*F*^2^) is used only for calculating *R*-factors(gt) *etc*. and is not relevant to the choice of reflections for refinement. *R*-factors based on *F*^2^ are statistically about twice as large as those based on *F*, and *R*- factors based on ALL data will be even larger.

### Fractional atomic coordinates and isotropic or equivalent isotropic displacement parameters (Å^2^)

**Table:**

	*x*	*y*	*z*	*U*_iso_*/*U*_eq_	
I2	1.04050 (3)	0.30244 (3)	−0.04759 (3)	0.06234 (11)	
I1	0.58853 (3)	0.01728 (3)	−0.20478 (3)	0.07768 (13)	
Cd1	0.80005 (3)	0.17348 (3)	−0.02843 (3)	0.05367 (11)	
S1	0.66738 (13)	0.72678 (11)	0.58132 (11)	0.0647 (3)	
S2	0.83801 (13)	0.63432 (12)	0.62502 (11)	0.0666 (3)	
N1	0.6891 (4)	0.3069 (3)	0.0542 (3)	0.0487 (9)	
N2	0.6043 (3)	0.5841 (3)	0.3679 (3)	0.0466 (8)	
N3	0.4190 (4)	0.6770 (3)	0.4080 (4)	0.0593 (10)	
C6	0.5105 (4)	0.5314 (3)	0.2591 (4)	0.0426 (10)	
C4	0.7136 (4)	0.4798 (4)	0.2009 (4)	0.0526 (11)	
H4A	0.7736	0.5465	0.2565	0.063*	
C9	0.5529 (4)	0.6526 (4)	0.4357 (4)	0.0508 (11)	
C8	0.3282 (5)	0.6238 (4)	0.2986 (5)	0.0618 (13)	
H8A	0.2334	0.6379	0.2739	0.074*	
C3	0.7684 (4)	0.4046 (4)	0.1332 (4)	0.0523 (11)	
H3B	0.8655	0.4238	0.1437	0.063*	
C5	0.5695 (4)	0.4544 (3)	0.1849 (4)	0.0423 (10)	
C2	0.5492 (5)	0.2843 (4)	0.0378 (4)	0.0632 (13)	
H2B	0.4911	0.2178	−0.0191	0.076*	
C1	0.4868 (5)	0.3542 (4)	0.1005 (4)	0.0606 (13)	
H1A	0.3889	0.3343	0.0864	0.073*	
C7	0.3683 (4)	0.5492 (4)	0.2207 (4)	0.0530 (11)	
H7A	0.3026	0.5122	0.1454	0.064*	
N5	0.9147 (3)	0.7871 (3)	0.4735 (3)	0.0474 (8)	
C13	1.0157 (4)	0.8354 (4)	0.4271 (4)	0.0493 (11)	
C14	0.9704 (4)	0.9207 (4)	0.3321 (4)	0.0459 (10)	
C10	0.9551 (5)	0.7070 (4)	0.5546 (4)	0.0571 (12)	
N4	1.0789 (5)	0.6629 (4)	0.5948 (4)	0.0815 (13)	
C18	1.0671 (4)	0.9895 (4)	0.2893 (4)	0.0548 (12)	
H18A	1.1651	0.9878	0.3252	0.066*	
C15	0.8267 (5)	0.9313 (4)	0.2767 (5)	0.0637 (13)	
H15A	0.7576	0.8889	0.3036	0.076*	
C12	1.1494 (5)	0.7979 (5)	0.4649 (5)	0.0737 (14)	
H12A	1.2198	0.8306	0.4345	0.088*	
C11	1.1754 (6)	0.7115 (6)	0.5484 (5)	0.0934 (18)	
H11A	1.2650	0.6856	0.5736	0.112*	
C17	1.0191 (5)	1.0597 (4)	0.1950 (5)	0.0566 (12)	
H17A	1.0864	1.1044	0.1676	0.068*	
N6	0.8813 (4)	1.0678 (3)	0.1399 (3)	0.0537 (9)	
C16	0.7870 (5)	1.0040 (4)	0.1829 (5)	0.0697 (14)	
H16A	0.6900	1.0097	0.1470	0.084*	

### Atomic displacement parameters (Å^2^)

**Table:**

	*U*^11^	*U*^22^	*U*^33^	*U*^12^	*U*^13^	*U*^23^
I2	0.04535 (17)	0.0650 (2)	0.0747 (2)	−0.00692 (14)	0.02573 (16)	−0.00033 (16)
I1	0.04633 (19)	0.0866 (2)	0.0967 (3)	−0.00948 (16)	0.03348 (19)	−0.0232 (2)
Cd1	0.04412 (19)	0.05401 (19)	0.0674 (3)	0.00516 (14)	0.02683 (18)	0.00403 (16)
S1	0.0556 (7)	0.0817 (8)	0.0595 (9)	0.0022 (6)	0.0296 (7)	−0.0090 (7)
S2	0.0583 (8)	0.0876 (9)	0.0536 (9)	0.0064 (6)	0.0201 (6)	0.0158 (7)
N1	0.043 (2)	0.051 (2)	0.053 (2)	0.0078 (17)	0.0185 (18)	0.0044 (18)
N2	0.0358 (19)	0.053 (2)	0.053 (2)	0.0038 (16)	0.0198 (18)	0.0010 (17)
N3	0.048 (2)	0.063 (2)	0.073 (3)	0.0126 (19)	0.028 (2)	0.005 (2)
C6	0.036 (2)	0.040 (2)	0.053 (3)	0.0036 (18)	0.017 (2)	0.009 (2)
C4	0.043 (2)	0.056 (3)	0.056 (3)	0.003 (2)	0.016 (2)	−0.002 (2)
C9	0.043 (3)	0.053 (2)	0.059 (3)	0.003 (2)	0.024 (2)	0.007 (2)
C8	0.042 (3)	0.066 (3)	0.085 (4)	0.019 (2)	0.028 (3)	0.014 (3)
C3	0.032 (2)	0.066 (3)	0.059 (3)	0.002 (2)	0.020 (2)	0.005 (2)
C5	0.037 (2)	0.044 (2)	0.049 (3)	0.0089 (18)	0.019 (2)	0.010 (2)
C2	0.043 (3)	0.063 (3)	0.076 (4)	−0.003 (2)	0.020 (3)	−0.019 (3)
C1	0.041 (2)	0.064 (3)	0.074 (4)	0.002 (2)	0.021 (2)	−0.008 (3)
C7	0.043 (3)	0.059 (3)	0.053 (3)	0.009 (2)	0.011 (2)	0.004 (2)
N5	0.0379 (19)	0.061 (2)	0.042 (2)	0.0099 (16)	0.0132 (17)	0.0014 (18)
C13	0.045 (2)	0.055 (2)	0.050 (3)	0.007 (2)	0.021 (2)	−0.009 (2)
C14	0.038 (2)	0.054 (2)	0.047 (3)	0.0098 (19)	0.018 (2)	−0.004 (2)
C10	0.045 (3)	0.077 (3)	0.050 (3)	0.014 (2)	0.016 (2)	0.002 (3)
N4	0.066 (3)	0.125 (4)	0.070 (3)	0.038 (3)	0.033 (3)	0.037 (3)
C18	0.036 (2)	0.061 (3)	0.068 (4)	0.004 (2)	0.022 (2)	0.000 (2)
C15	0.040 (3)	0.087 (3)	0.075 (4)	0.008 (2)	0.031 (3)	0.024 (3)
C12	0.047 (3)	0.113 (4)	0.076 (4)	0.027 (3)	0.033 (3)	0.024 (3)
C11	0.059 (3)	0.154 (6)	0.087 (5)	0.052 (4)	0.034 (3)	0.046 (4)
C17	0.043 (3)	0.054 (3)	0.077 (4)	0.001 (2)	0.029 (3)	0.003 (3)
N6	0.046 (2)	0.054 (2)	0.069 (3)	0.0120 (17)	0.028 (2)	0.0135 (18)
C16	0.038 (3)	0.091 (4)	0.092 (4)	0.017 (2)	0.033 (3)	0.034 (3)

### Geometric parameters (Å, °)

**Table:**

I2—Cd1	2.6962 (4)	C2—H2B	0.9300
I1—Cd1	2.6938 (5)	C1—H1A	0.9300
Cd1—N1	2.287 (3)	C7—H7A	0.9300
Cd1—N6^i^	2.290 (4)	N5—C10	1.314 (5)
S1—C9	1.771 (4)	N5—C13	1.360 (5)
S1—S2	2.0205 (19)	C13—C12	1.383 (6)
S2—C10	1.774 (4)	C13—C14	1.470 (6)
N1—C3	1.326 (5)	C14—C18	1.383 (5)
N1—C2	1.334 (5)	C14—C15	1.386 (6)
N2—C9	1.325 (4)	C10—N4	1.330 (6)
N2—C6	1.338 (5)	N4—C11	1.325 (6)
N3—C9	1.334 (5)	C18—C17	1.359 (6)
N3—C8	1.336 (5)	C18—H18A	0.9300
C6—C7	1.383 (5)	C15—C16	1.362 (6)
C6—C5	1.480 (5)	C15—H15A	0.9300
C4—C5	1.378 (5)	C12—C11	1.370 (7)
C4—C3	1.388 (5)	C12—H12A	0.9300
C4—H4A	0.9300	C11—H11A	0.9300
C8—C7	1.376 (5)	C17—N6	1.326 (5)
C8—H8A	0.9300	C17—H17A	0.9300
C3—H3B	0.9300	N6—C16	1.343 (5)
C5—C1	1.381 (5)	N6—Cd1^ii^	2.290 (4)
C2—C1	1.367 (5)	C16—H16A	0.9300
			
N1—Cd1—N6^i^	96.12 (11)	C5—C1—H1A	120.0
N1—Cd1—I1	106.24 (9)	C8—C7—C6	117.1 (4)
N6^i^—Cd1—I1	108.88 (8)	C8—C7—H7A	121.4
N1—Cd1—I2	109.95 (8)	C6—C7—H7A	121.4
N6^i^—Cd1—I2	104.57 (8)	C10—N5—C13	115.5 (4)
I1—Cd1—I2	126.810 (15)	N5—C13—C12	119.8 (4)
C9—S1—S2	104.45 (14)	N5—C13—C14	116.3 (4)
C10—S2—S1	106.08 (18)	C12—C13—C14	123.7 (4)
C3—N1—C2	117.0 (3)	C18—C14—C15	116.6 (4)
C3—N1—Cd1	119.2 (2)	C18—C14—C13	122.1 (4)
C2—N1—Cd1	123.3 (3)	C15—C14—C13	121.3 (4)
C9—N2—C6	116.3 (3)	N5—C10—N4	129.3 (4)
C9—N3—C8	114.5 (3)	N5—C10—S2	121.9 (3)
N2—C6—C7	121.2 (3)	N4—C10—S2	108.7 (4)
N2—C6—C5	115.6 (3)	C11—N4—C10	114.0 (5)
C7—C6—C5	123.3 (4)	C17—C18—C14	120.1 (4)
C5—C4—C3	119.2 (4)	C17—C18—H18A	120.0
C5—C4—H4A	120.4	C14—C18—H18A	120.0
C3—C4—H4A	120.4	C16—C15—C14	119.9 (4)
N2—C9—N3	127.8 (4)	C16—C15—H15A	120.1
N2—C9—S1	120.2 (3)	C14—C15—H15A	120.1
N3—C9—S1	112.0 (3)	C11—C12—C13	118.2 (4)
N3—C8—C7	123.1 (4)	C11—C12—H12A	120.9
N3—C8—H8A	118.4	C13—C12—H12A	120.9
C7—C8—H8A	118.4	N4—C11—C12	123.1 (5)
N1—C3—C4	123.3 (4)	N4—C11—H11A	118.5
N1—C3—H3B	118.4	C12—C11—H11A	118.5
C4—C3—H3B	118.4	N6—C17—C18	123.5 (4)
C4—C5—C1	117.2 (3)	N6—C17—H17A	118.2
C4—C5—C6	119.8 (4)	C18—C17—H17A	118.2
C1—C5—C6	122.9 (3)	C17—N6—C16	116.9 (4)
N1—C2—C1	123.2 (4)	C17—N6—Cd1^ii^	123.1 (3)
N1—C2—H2B	118.4	C16—N6—Cd1^ii^	119.9 (3)
C1—C2—H2B	118.4	N6—C16—C15	123.0 (4)
C2—C1—C5	120.0 (4)	N6—C16—H16A	118.5
C2—C1—H1A	120.0	C15—C16—H16A	118.5
			
C9—S1—S2—C10	84.1 (2)	N3—C8—C7—C6	−1.0 (6)
N6^i^—Cd1—N1—C3	76.8 (3)	N2—C6—C7—C8	0.5 (6)
I1—Cd1—N1—C3	−171.5 (3)	C5—C6—C7—C8	−179.6 (4)
I2—Cd1—N1—C3	−31.2 (3)	C10—N5—C13—C12	0.9 (6)
N6^i^—Cd1—N1—C2	−95.6 (4)	C10—N5—C13—C14	177.3 (3)
I1—Cd1—N1—C2	16.2 (4)	N5—C13—C14—C18	171.0 (3)
I2—Cd1—N1—C2	156.5 (3)	C12—C13—C14—C18	−12.8 (6)
C9—N2—C6—C7	0.4 (5)	N5—C13—C14—C15	−12.1 (5)
C9—N2—C6—C5	−179.5 (3)	C12—C13—C14—C15	164.2 (4)
C6—N2—C9—N3	−1.0 (6)	C13—N5—C10—N4	−2.6 (7)
C6—N2—C9—S1	−180.0 (3)	C13—N5—C10—S2	−180.0 (3)
C8—N3—C9—N2	0.6 (6)	S1—S2—C10—N5	−11.3 (4)
C8—N3—C9—S1	179.6 (3)	S1—S2—C10—N4	170.8 (3)
S2—S1—C9—N2	−18.5 (3)	N5—C10—N4—C11	2.6 (8)
S2—S1—C9—N3	162.4 (3)	S2—C10—N4—C11	−179.7 (4)
C9—N3—C8—C7	0.4 (6)	C15—C14—C18—C17	−2.1 (6)
C2—N1—C3—C4	2.3 (6)	C13—C14—C18—C17	175.0 (4)
Cd1—N1—C3—C4	−170.5 (3)	C18—C14—C15—C16	1.9 (6)
C5—C4—C3—N1	−1.2 (6)	C13—C14—C15—C16	−175.2 (4)
C3—C4—C5—C1	−0.3 (6)	N5—C13—C12—C11	0.5 (7)
C3—C4—C5—C6	178.2 (4)	C14—C13—C12—C11	−175.7 (4)
N2—C6—C5—C4	−26.6 (5)	C10—N4—C11—C12	−0.9 (8)
C7—C6—C5—C4	153.5 (4)	C13—C12—C11—N4	−0.4 (9)
N2—C6—C5—C1	151.8 (4)	C14—C18—C17—N6	0.6 (6)
C7—C6—C5—C1	−28.1 (6)	C18—C17—N6—C16	1.2 (6)
C3—N1—C2—C1	−2.0 (7)	C18—C17—N6—Cd1^ii^	−175.0 (3)
Cd1—N1—C2—C1	170.5 (4)	C17—N6—C16—C15	−1.4 (7)
N1—C2—C1—C5	0.6 (7)	Cd1^ii^—N6—C16—C15	174.9 (4)
C4—C5—C1—C2	0.6 (6)	C14—C15—C16—N6	−0.1 (7)
C6—C5—C1—C2	−177.9 (4)		

Symmetry codes: (i) *x*, *y*−1, *z*; (ii) *x*, *y*+1, *z*.
